# Biogenic Amines in Plant-Origin Foods: Are they Frequently Underestimated in Low-Histamine Diets?

**DOI:** 10.3390/foods7120205

**Published:** 2018-12-14

**Authors:** Sònia Sánchez-Pérez, Oriol Comas-Basté, Judit Rabell-González, M. Teresa Veciana-Nogués, M. Luz Latorre-Moratalla, M. Carmen Vidal-Carou

**Affiliations:** 1 Departament de Nutrició, Ciències de l’Alimentació i Gastronomia, Facultat de Farmàcia i Ciències de l’Alimentació, Universitat de Barcelona (UB), Av. Prat de la Riba 171, 08921 Santa Coloma de Gramenet, Spain; soniasanchezperez@ub.edu (S.S.-P.); oriolcomas@ub.edu (O.C.-B.); juditrabellgonzalez@hotmail.com (J.R.-G.); veciana@ub.edu (M.T.V.-N.); mariluzlatorre@ub.edu (M.L.L.-M.); 2 Institut de Recerca en Nutrició i Seguretat Alimentària (INSA·UB), Universitat de Barcelona (UB), Av. Prat de la Riba 171, 08921 Santa Coloma de Gramenet, Spain; 3 Xarxa de Referència en Tecnologia dels Aliments de la Generalitat de Catalunya (XaRTA), C/ Baldiri Reixac 4, 08028 Barcelona, Spain

**Keywords:** histamine, putrescine, tyramine, cadaverine, biogenic amines, histamine intolerance, low-histamine diet, plant-origin foods, culinary process, storage conditions

## Abstract

Low-histamine diets are currently used to reduce symptoms of histamine intolerance, a disorder in histamine homeostasis that increases plasma levels, mainly due to reduced diamine-oxidase (DAO) activity. These diets exclude foods, many of them of plant origin, which patients associate with the onset of the symptomatology. This study aimed to review the existing data on histamine and other biogenic amine contents in nonfermented plant-origin foods, as well as on their origin and evolution during the storage or culinary process. The only plant-origin products with significant levels of histamine were eggplant, spinach, tomato, and avocado, each showing a great variability in content. Putrescine has been found in practically all plant-origin foods, probably due to its physiological origin. The high contents of putrescine in certain products could also be related to the triggering of the symptomatology by enzymatic competition with histamine. Additionally, high spermidine contents found in some foods should also be taken into account in these diets, because it can also be metabolized by DAO, albeit with a lower affinity. It is recommended to consume plant-origin foods that are boiled or are of maximum freshness to reduce biogenic amine intake.

## 1. Introduction

In recent years, various diets have been proposed for the treatment of histamine intolerance [1,2,3,4,5,6,7,8]. These diets, known as low- or free-histamine diets, usually exclude foods that patients associate with the onset of intolerance symptoms. Such foods tend to be rich in histamine, but some, surprisingly, are not usually regarded as sources of this amine.

As described in the literature and scientific reports issued by the European Food Safety Authority (EFSA) and a joint Food and Agriculture Organization of the United Nations (FAO)/World Health Organization (WHO) committee, histamine intolerance (also called food histaminosis or food histamine sensitivity) is a disorder associated with increased plasma histamine levels and is recognized as clinically different from the more established histamine intoxication [9,10]. Although in both cases, histamine is the causative agent, the etiology of the disorders differs. Intoxication appears after the consumption of foods with unusually high histamine concentrations, while intolerance is due to a deficiency in histamine metabolism, so that symptoms may be triggered even by the intake of low amounts [1,9,10,11].

Diamine oxidase (DAO) is the main enzyme responsible for the metabolism of histamine and other amines at the intestinal level, and impaired DAO activity is one of the main causes of histamine intolerance [1,12,13]. This enzymatic deficit may have its origins in genetic mutations. Different polymorphisms of a single nucleotide in the gene that encodes this enzyme (*AOC1* on chromosome 7) have been associated with lower DAO activity [14,15,16]. The deficit may also be due to acquired causes such as inflammatory bowel diseases that block the secretion of DAO [1,3,12], or to the inhibitory action of drugs, some of them with a very widespread use (e.g., acetylcysteine, clavulanic acid, metoclopramide, verapamil) [1,17]. Another enzyme involved in histamine metabolization is monoamine oxidase (MAO) [13]. Therefore, MAO inhibitor drugs, such as selegiline or rasagiline, could also favor the plasmatic accumulation of histamine and the onset of symptoms of histamine intolerance. In addition, the presence of other biogenic amines, mainly putrescine and cadaverine, may compromise the intestinal degradation of histamine by enzymatic competition with DAO [9].

The symptoms of histamine intolerance are numerous and highly variable, due to the effects and functions of histamine in multiple organs and systems of the body. They include gastrointestinal (abdominal pain, diarrhea, vomiting), dermatological (urticaria, dermatitis, or pruritus), respiratory (rhinitis, nasal congestion, and asthma), cardiovascular (hypotonia and arrhythmias), and neurological (headaches) symptoms, and it is common for more than one disorder to occur simultaneously [1,11,12]. Several clinical studies have shown that patients with a potential diagnosis of histamine intolerance or with a diagnosis of migraine, intestinal, or dermatological diseases (atopic dermatitis, eczema, or chronic urticaria) have a higher prevalence of DAO deficits compared to the control population [3,6,18,19,20,21,22,23,24,25,26,27,28].

In order to carry out a correct dietary treatment of histamine intolerance, it is necessary to know what foods may contain this amine and what factors influence its accumulation. Likewise, it is also important to consider the occurrence of other amines that are also metabolized by the DAO enzyme. In contrast to plant-origin foods, there is more available information on the contents of histamine and other amines in fish and fish derivatives and all types of fermented products (cheeses, sausages, sauerkraut, wines, beer), in which their presence is attributed to the aminogenic activity of spoilage microorganisms and also to fermentative microorganisms [9,10,29]. Therefore, the freshness of the food and the hygienic conditions of the raw materials and manufacturing processes, as well as the adequate selection of starter cultures without decarboxylase activity, are of vital importance to avoid or reduce the formation of these compounds [9,29,30,31].

Due to the information available on the contents of biogenic amines in nonfermented plant-origin foods being scarce, the aim of this study was to review the existing data on the contents of histamine and other biogenic amines in these types of products, as well as their origin and evolution during storage or cooking.

## 2. Methods

A selective search of scientific literature dealing with biogenic amine contents in nonfermented plant-origin foods, including vegetables, fruits, and cereals, was performed. The bibliographic search was carried out in the PubMed and Web of Science databases using the following keywords: “histamine”, “biogenic amines”, “tyramine”, “putrescine”, “cadaverine”, “plant-origin food”, “food samples”, “storage”, “cooking”, “fruit”, “vegetable”, “legume”, “cereal”, “spinach”, “eggplant”, “tomato”, “citrus”, “modified atmosphere packaging”, and “microbial decarboxylase activity”. Original analytical studies, reviews, and table compilations of content in food were included. Articles published before 1990 were excluded from this review. 

Apart from data obtained from the literature, data on the biogenic amine content of plant-origin foods from our own database of Spanish market products were also used. Specifically, histamine, tyramine, putrescine, and cadaverine contents of 25 types of vegetables, 19 fruits, and 8 cereals were included.

## 3. Content of Biogenic Amines in Plant-Origin Foods 

In this section, the contents of biogenic amines (histamine, tyramine, putrescine, and cadaverine) in different plant-origin foods are reviewed, using our own database and data from studies published by other authors. A total of 20 studies reporting data on biogenic amine contents in such foods were found. Most provided data on putrescine contents (normally together with the polyamines spermine and spermidine, not dealt with in this section), and only a few included other amines, such as histamine, tyramine, and cadaverine.

### 3.1. Vegetables and Legumes

Table 1 shows the contents of biogenic amines in different types of vegetables and legumes (nonfermented).

The only products found to contain significant levels of histamine were eggplant, spinach, and tomato, each showing a great variability in content, both in samples from the same study and among different studies. Histamine values ranged from 4.2 to 100.6 mg/kg in eggplant, from 9.5 to 69.7 mg/kg in spinach, and from not detected to 17.1 mg/kg in tomato. In the case of asparagus, pumpkin, and chard, histamine was found in only a few samples and at very low levels (<2 mg/kg).

Histamine occurs naturally in certain foods [29,32], which explains why it was recorded in practically all samples of spinach, eggplant, and tomato. The variability observed may have been due to botanical variety, as reported by Kumar et al. [33] for eggplant. However, as occurs in foods of animal origin, the presence of high contents of histamine and other amines in plant-origin products could also be associated with microbial activity [29,32,34]. Lavizzari et al. [32] attributed the high contents of histamine in spinach to the activity of contaminating bacteria during storage, belonging mainly to the groups Enterobacteriaceae and Pseudomonadaceae. There is currently a need for more research to understand in more detail the origin of histamine in plant foods such as spinach, eggplant, and tomatoes.

Tyramine has been found in more foods than histamine, although in lower concentrations, in no case exceeding 10 mg/kg. It should be noted that histamine-containing foods also contained tyramine (eggplants, tomatoes, spinach, chard, and asparagus). Although there is very little information about the origin of tyramine in nonfermented vegetables, its presence seems to be associated with microbial aminogenic activity. The ability to form tyramine has been reported for bacteria of the genus *Enterococcus* isolated from plants and fruits, mainly *E. faecium*, *E. mundtii*, and *E. casseliflavus* [35].

Putrescine has been detected in all the studied vegetables and legumes, although its content varied greatly among foods and sometimes also within the same product. In most vegetables and legumes, the average values ranged from 1 to 25 mg/kg. However, some samples of green pepper, eggplant, sweet corn, green and purple beans, spinach, tomato ketchup, soybeans, and peas had strikingly high putrescine contents, in some cases exceeding 200 mg/kg (Table 1). The putrescine found in food can have a dual origin. In plant-origin foods, low contents of this amine generally have a physiological source, as it performs different functions in plants, as do the polyamines spermidine and spermine, ranging from the activation of organogenesis to protection against stress [34,36,37]. On the other hand, the presence of putrescine is also associated with the decarboxylase activity of different groups of spoilage bacteria, mainly Enterobacteriaceae and *Clostridium* spp. [36]. According to Kalač et al. [38], the high amounts of putrescine found in frozen peas are due to bacterial activity in the period between harvesting and freezing or during thawing. However, high putrescine contents cannot always be attributed to bacterial decarboxylase activity. Toro-Funes et al. [39] have suggested that the considerable levels of putrescine found in soybean sprouts arise from the germination process, as this amine is a plant growth factor. In general, based on the available information, and due to the great variability in the reported contents, it is difficult to establish to what degree the presence of putrescine in plant-origin products can be considered physiological or the result of bacterial activity.

Cadaverine, like tyramine, has been described in few vegetables and legumes and in relatively low concentrations, with average values that in no case exceeded 8 mg/kg. The values reported by Nishimura et al. [40] in onion (29 mg/kg) and tofu (18 mg/kg) were an exception.

### 3.2. Fruits and Nuts

Table 2 shows the content of biogenic amines in different types of fresh fruits, fruit juices, and nuts. There were fewer publications reporting amine data for this type of food than for vegetables and legumes. In general, the contents were low, putrescine being in many cases the only amine found (in addition to the polyamines spermidine and spermine).

Avocado and kiwi, and grapefruit, orange, and pineapple juices, are the only products in this category for which the presence of histamine has been reported, but not in all studies. The 23 mg/kg of histamine in avocado reported by Jarisch et al. [12] stands out, although no relevant information about its possible origin was provided. A study conducted by Preti et al. [41] concluded that the presence of histamine in grapefruit, orange, and pineapple juices is due to a lack of hygienic quality during processing or storage, since this amine is not found in the original fresh fruit.

Similarly, very few fruits contained tyramine, and levels have always been low (Table 2). Avocado and plum stand out for their content of this amine, although in no case has it exceeded 7 mg/kg.

Putrescine has been found in practically all the fruits and nuts, with the highest levels in orange, orange juice, mandarin, grapefruit, grapefruit juice, banana, passion fruit, and pistachio. The range of contents of this amine in citrus fruits and their juices has been very broad, varying from not detected to as high as 200 mg/kg. Suggested explanations for this variability have included different origins, cultivation, and transport and storage conditions [41,42,43,44]. As reported by Gonzalez-Aguilar et al. [45], the contents of putrescine in mandarin (flavedo) can be increased by a drop in temperature before harvesting and by damage of mechanical origin. Its presence in most of the samples, unaccompanied by high levels of other amines (related to bacterial activity), seemed to indicate that, with some exceptions, putrescine in fruits has a physiological origin. To confirm this, it would be necessary to carry out more studies analyzing the fruit at the moment of collection. The only fruits reported as having no putrescine were avocado and plum, although interestingly, these did contain histamine and tyramine.

The only fruits with a notable content of cadaverine were bananas and sunflower seeds, for which Nishimura et al. [40] reported average levels of 11 and 22 mg/kg, respectively, although these data were from the analysis of only two samples. 

### 3.3. Cereals and Derivatives

Table 3 shows the contents of biogenic amines in cereals and some derivatives such as breakfast cereals, pasta, and bread. The quantitative information available on amines in cereals is very limited. In principle, these foods do not contain amines other than putrescine, which has a physiological origin [36]. The only standout source of putrescine is wheat germ, which, like soya bean sprouts, has a high rate of cell division, in which putrescine and polyamines play a significant role [36]. 

Putrescine contents in wholemeal bread were slightly higher than in bread made with refined flour. In white bread, low contents of cadaverine have also been reported, although only in one study, and from the analysis of two samples.

## 4. Evolution of Amine Contents during Storage and Cooking

The variability of amine contents observed among samples of the same product can be attributed mainly to conditions of production, transport, and storage [42].

The storage temperature is one of the most important factors in the formation of biogenic amines [11,29]. Refrigeration delays or reduces the aminogenic potential of microorganisms, although the formation of amines at refrigeration temperatures (4–10 °C) has been reported. The influence of the conservation temperature has been widely studied in foods such as meats, fish, and fermented products [29,30,58], but scarcely in plant-origin foods.

A study conducted by Simon-Sarkadi et al. [59] showed a clear increase in putrescine in different types of green leafy vegetables (lettuce, endives, Chinese cabbage, and radicchio) during six days of storage at 5 °C. The authors concluded that there was a positive correlation between putrescine contents and the hygienic state of these foods (total microorganism counts). Tyramine contents also showed a tendency to increase slightly. Histamine was present only in Chinese cabbage and in very low concentrations, remaining stable throughout the study period. In contrast, when Moret et al. [34] studied the effect of storage temperature on the amine content in various vegetables (parsley, zucchini, broccoli, and cucumber), no significant changes in histamine, tyramine, putrescine, and cadaverine were observed after three weeks of refrigeration.

Lavizzari et al. [32] also reported an increase in histamine in different spinach samples over 12–15 days of storage at 6 °C, noting that the relatively high pH of this vegetable favored the growth of Gram-negative bacteria, which could have been responsible for the formation of this amine during storage. The contents of tyramine and putrescine did not undergo significant changes under these storage conditions. It should be noted that in two of the five trials carried out in this study, histamine levels decreased in the last days of storage. The authors suggested that this histamine degradation could have been due to the action of bacteria with DAO activity, as well as the effect of the pH, which reached values above 8 [32]. Another study also recently reported the complete degradation of histamine in a spinach sample (61 mg/kg) after three weeks of storage at 4 °C [54].

Modified atmosphere packaging, together with low storage temperatures, is commonly used to extend the life of fresh vegetables and fruits. This type of packaging can influence the capacity of microorganisms to form amines [30,58,60]. Esti et al. [43] monitored the contents of amines during the ripening of cherries and apricots packaged in modified atmospheres and stored at 0 °C, and found that after 20 days of storage the contents of amines (mainly putrescine) had decreased by 20% compared to the initial value. Although the authors did not provide an explanation for this reduction, it could have been due to putrescine serving as a substrate for polyamine formation [36].

Another factor that can affect the content of biogenic amines in foods of plant origin, especially vegetables, is the culinary process. Again, the results reported in the literature were variable, depending on the type of cooking and the amine in question. 

Latorre-Moratalla et al. [61] evaluated the effect of cooking spinach in water, with or without salt. The cooking process reduced the histamine content in all the samples by an average of 83% with respect to the raw product (after a correction for the dilution effect of the cooking). Analysis confirmed a transfer of histamine to the cooking water, which was not enhanced by the addition of salt. Likewise, Kumar et al. [33] observed the loss of 11–14% histamine in eggplants boiled at 100 °C for 10 min. Veciana et al. [62] also concluded that the putrescine content in certain vegetables (spinach, cauliflower, Swiss chard, potato, and green beans) is reduced by transfer to the cooking water. However, this heat treatment had no effect on the putrescine content in other vegetables such as pepper, pea, and asparagus. Eliassen et al. [42] also found no significant differences in putrescine levels among different types of raw and boiled vegetables (carrot, broccoli, cauliflower, and potato), although they acknowledged that the low number of samples analyzed (two per food) was a limitation when trying to reach a conclusion.

Conversely, three recent studies have shown an increase in amine levels after a cooking process. According to Lo Scalzo et al. [63], boiling and grilling enhanced the putrescine content in a specific variety of eggplant by 55% and 32%, respectively. In the other two varieties of eggplant tested, the cooking had no effect. Similarly, Preti et al. [46] reported a significant increase in putrescine in green beans after boiling, whereas steaming did not modify the contents. According to the work performed by Chung et al. [64], frying brought about a 2.5- and 4-fold increase in histamine in carrots and seaweed, respectively. The authors attributed this increase to the loss of water caused by the high heat treatment. The same process had no effect on spinach and onions. However, it should be noted that in this study, the contents of histamine in all foods were well below 1 mg/kg, both before and after frying.

Amines are thermostable compounds, so in principle changes in contents can only be due to their transfer to the cooking water or by dilution or concentration effects of the culinary process, in which the food gains or loses water.

## 5. Plant-Origin Foods in Low-Histamine Diets

At present, the main strategy to prevent the onset of histamine intolerance symptoms is to follow a low-histamine diet. Its efficacy has been demonstrated in different clinical studies, which have always described an improvement or remission of gastrointestinal, dermatological, and neurological symptoms [3,6,18,19,20,22,24,27,65,66,67] if the diet was followed. 

Current low-histamine diets exclude foods that patients associate with the onset of symptoms [1,2,3,4,5,6,7,8], such as blue fish and their preserves, and all kinds of fermented products (cheeses, sausages, wine, beer, sauerkraut, and fermented soy derivatives), all of which are susceptible to having high contents of histamine and other amines. A high number of nonfermented plant-origin foods are also excluded: The average contents of biogenic amines and polyamines in these foods are shown in Table 4. As can be seen, with the exception of spinach, eggplant, tomatoes, and avocado, for which high amounts of histamine have been described, the rest contained very little or no histamine, so a priori should not be responsible for triggering symptoms. However, some of them had relatively high contents of other biogenic amines and polyamines. 

Putrescine, cadaverine, and tyramine are all substrates of the DAO enzyme, so if present in high amounts they may increase the adverse effects of histamine by competing as rival substrates or for binding sites in the intestinal mucosa [1,9,68,69]. The high putrescine contents found in citrus fruits, mushrooms, soybeans, bananas, and nuts could thus explain why patients associate their consumption with the onset of histamine intolerance symptoms. However, it should be noted that some foods with similar or even much higher putrescine contents, such as green pepper, peas, or corn, are permitted in low-histamine diets (Table 1).

The polyamines spermidine and spermine can also be metabolized by DAO, albeit with a lower affinity [68,69], and therefore their presence should also be taken into account in this type of diet (Table 4). Thus, the exclusion of foods such as soybeans, mushrooms, lentils, chickpeas, peanuts, nuts, and pears may be justified by their high polyamine content.

Finally, the levels of biogenic amines and polyamines found in kiwi, papaya, strawberry, pineapple, and plum are too low to justify their exclusion. Some authors consider these foods, along with others such as milk, shellfish, and eggs, as endogenous histamine releasers, although by mechanisms still not well understood [1,11,70].

## 6. Conclusions

Biogenic amine data in nonfermented plant-origin foods from the different reviewed studies showed a great variability both within the same food item and among them. Putrescine was the most frequent biogenic amine found in fresh vegetables, legumes, fruits, and cereals, and only a limited number of products contained relevant levels of histamine (eggplant, spinach, tomato, and avocado). Tyramine and cadaverine were usually more scarcely found in plant-origin foods. Generally, low levels of histamine and putrescine may have a physiological origin. However, undesirable microbial enzymatic activity during production or storage may lead to the accumulation of high levels of these amines.

No single trend has emerged in the evolution of amine contents during refrigerated storage, which might be at least partly due to the different experimental designs of the studies. In some cases, refrigeration seems to have prevented the formation of certain amines, but this remains a hypothesis, as no study performed a comparative analysis of samples stored under refrigeration and at room temperature. The increase in the biogenic amine content during refrigerated storage reported by other authors may be attributed to bacterial activity. Additionally, some studies have observed an influence of culinary process on the biogenic amine content, mainly derived from the transfer of these compounds to the boiling water or by dilution or concentration effects of the applied treatment.

The exclusion of a high number of plant-origin foods from low-histamine diets cannot be accounted for by their histamine contents, but is more likely due to high levels of putrescine or spermidine. The plant-origin foods consumed by people with histamine intolerance should be of maximum freshness, since histamine and other amines may continue to form during refrigerated storage. The cooking of vegetables in water (boiling) is another relevant strategy for this population, since it can reduce the contents of histamine and other amines in the food.

## Figures and Tables

**Table 1:** Biogenic amine contents (mg/kg fresh weight) found in vegetables and legumes. Data are presented as average (standard deviation) and range (minimum–maximum).

Food Categories	*n*	Occurrence of Biogenic Amines (mg/kg)								Reference
		Histamine		Tyramine		Putrescine		Cadaverine		
		Mean (SD)	Range	Mean (SD)	Range	Mean (SD)	Range	Mean (SD)	Range	
**Vegetables and Vegetable Products**										
Asparagus (wheat)	5	0.34 (0.62)	nd–1.42	0.69 (0.88)	nd–2.1	13.08 (2.81)	8.58–16.27	0.12 (0.19)	nd–0.43	#
Beans (green)	12	nd	-	2.46 (3.24)	nd–9.86	10.30 (8.61)	2.98–28.81	nd	-	#
	-	nd	-	nd	-	34.9 (6.2)	-	-	-	[46]
Beans (purple)	-	nd	-	nd	-	77.8 (7.7)	-	nd	-	[46]
Beans (yellow)	-	nd	-	nd	-	14.8 (1.4)	-	nd	-	[46]
Broccoli	4	-	-	-	-	9	7–10.5	-	-	[47]
	5	-	-	-	-	6.4 (2.9)	3.4–10.8	-	-	[42]
	10	-	-	-	-	5.7	-	-	-	[48]
	5	-	-	-	-	1.9	1.3–2.6	-	-	[49]
	2	-	-	-	-	-	8.37–20.53	0.1	-	[40]
Cabbage	3	-	-	-	-	-	-	-	-	[47]
	-	-	-	<0.5	-	16	-	-	-	[34]
	2	-	-	-	-	14.18	-	6.03	-	[40]
Cabbage (white)	10	-	-	-	-	6.6	-	-	-	[48]
	8	-	-	-	-	2.7	0.7–6.2	-	-	[49]
Cauliflower	3	nd	-	1.05 (0.91)	nd–1.71	3 (1.49)	1.61–4.58	0.16 (0.27)	nd–0.48	#
	3	-	-	-	-	-	3.1–4.5	-	-	[50]
	7	-	-	-	-	4.9	2.2–7.6	-	-	[47]
	5	-	-	-	-	5.3 (2.1)	3.3–8.9	-	-	[42]
	2	-	-	-	-	3.7	-	-	-	[40]
	-	-	-	<0.5	-	9	-	<0.5		[34]
Carrot	13	nd	-	nd	-	2.27 (2.20)	0.35–8.92	nd	-	#
	3	-	-	-	-	-	1.2–1.8	-	-	[50]
	4	-	-	-	-	2.8	2–3.9	-	-	[47]
	2	-	-	-	-	3.5	-	-	-	[51]
	6	-	-	-	-	1.5 (0.7)	0.7–2.7	-	-	[42]
	10	-	-	-	-	0.7	-	-	-	[48]
	4	-	-	-	-	12.1 (3.9)	7–18	-	-	[52]
	5	-	-	-	-	14.8	8–24.7	-	-	[49]
	-	-	-	1	-	5	-	-	-	[34]
	2	-	-	-	-	-	5.73–14.10	2.55	-	[40]
Celeriac	3	-	-	-	-	6.1	3.7–7.7	-	-	[47]
Chard	8	0.79 (0.41)	nd–1.33	1.90 (0.98)	0.74–3.48	6.38 (3.22)	2.4–11.94	0.13 (0.11)	nd–0.24	#
Courgette	41	nd	-	nd	-	7.94 (4.12)	2.74–24.81	0.32 (0.7)	nd–2.07	#
	-	-	-	2	-	4	-	-	-	[34]
Cucumber	10	nd	-	0.61 (0.89)	nd–2.33	5.42 (3.13)	1.32–10.62	nd	-	#
	3	-	-	-	-	3.2	-	-	-	[50]
	5	-	-	-	-	6.9 (1.4)	5.5–8.7	-	-	[42]
	10	-	-	-	-	8.7	-	-	-	[48]
	5	-	-	-	-	13.1	2.1–28.8	-	-	[49]
	-	-	-	1	-	25	-	-	-	[34]
	2	-	-	-	-	9.87	-	2.96	-	[40]
Eggplant	23	39.42 (30.66)	4.17–100.6	0.60 (0.90)	nd–2.27	34.30 (6.98)	24.10–48.63	nd	-	#
	-	26	-	-	-	-	-	-	-	[12]
	2	-	-	-	-	18.35	-	5	-	[40]
Lettuce	4	nd	-	nd	-	2.85 (0.75)	2.20–3.90	nd	-	#
	3	-	-	-	-	-	3.3–4.8	-	-	[50]
	3	-	-	-	-	5.6 (1.3)	4.5–7.3	-	-	[42]
	10	-	-	-	-	7.9	-	-	-	[48]
	7	-	-	-	-	20.7	10.2–42.3	-	-	[49]
	2	-	-	-	-	6.87	-	3.27	-	[40]
Mushroom	11	nd	-	nd	-	1.29 (1.20)	0.02–3.65	0.10 (0.4)	nd–1.59	#
	10	-	-	-	-	11.7	-	-	-	[48]
	213	-	-	-	-	-	nd–156	-	-	[53]
Onion	4	nd	-	nd	-	nd	-	nd	-	#
	3	-	-	-	-		5.5–7.2	-	-	[50]
	10	-	-	-	-	0.5	-	-	-	[48]
	6	-	-	-	-	0.6	0.2–1	-	-	[49]
	-	-	-	3	-	2	-		-	[34]
	2	-	-	-	-	3.96	-	29.32	-	[40]
Pepper (green)	9	nd	-	nd	-	90.04 (41.65)	11.7–148.9	0.05 (0.14)	nd–0.41	#
	2	-	-	-	-	-	104–237	5.62	-	[40]
	5	-	-	-	-	70 (31)	13.2–96.9	-	-	[52]
Pepper (red)	8	nd	-	nd	-	2.42 (2.21)	0.59–5.35	nd	-	#
Potato	10	nd	-	0.58 (0.64)	nd–2.2	4.14 (3.06)	1.05–11.68	0.22 (0.54)	nd–1.75	#
	3	-	-	-	-	9.7	-	-	-	[50]
	3	-	-	-	-	17.6	-	-	-	[51]
	6	-	-	-	-	9.7 (2.1)	5.8–12.8	-	-	[42]
	2	-	-	-	-	-	0.1–22.4	-	-	[40]
	10	-	-	-	-	2.8	-	-	-	[48]
	6	-	-	-	-	7.2	1.1–10.5	-	-	[49]
	-	-	-	5	-	8	-	<0.5	-	[34]
Pumpkin	12	0.28 (0.54)	nd–1.90	nd	-	9.87 (6.19)	2.95–24.23	0.54 (0.76)	nd–2.15	#
Spinach	18	31.77 (17.02)	9.46–69.71	2.05 (0.83)	0.785–4.28	4.48 (2.46)	0.14–9.19	nd	-	#
	5	-	-	-	-	4.8	1.8–13.5	-	-	[49]
	-	16	-	6	-	6.0	-	1	-	[34]
	-	-	30–60	-	-	-	-	-	-	[12]
	-	37.5	-	-	-	-	-	-	-	[5]
	2	-	-	-	-	4.41	-	8.48	-	[40]
	-	61 (1.5)	-	nd	-	7.8 (0.1)	-	nd	-	[54]
	32	-	-	-	-	12.9	nd–119	-	-	[52]
Sweet corn	5	nd	-	nd	-	38.44 (9.50)	30.5–119.2	0.18 (0.25)	nd–0.46	#
Tomato	53	2.51 (4.08)	nd–17.07	0.49 (0.92)	nd–6.38	16.48 (6.93)	6.29–35.55	0.50 (0.48)	nd–2.33	#
	3	-	-	-	-	-	9.3–122	-	-	[50]
	2	-	-	-	-	10.6	-	-	-	[51]
	5	-	-	-	-	10	5.3–20.7	-	-	[49]
	-	6	-	<0.5	-	23	-	<0.5	-	[34]
	-	22	-	-	-	-	-	-	-	[5]
	2	-	-	-	-	23.96	-	1.63	-	[40]
Tomato (concentrated)	19	-	-	-	-	25.9 (8.2)	7.9–41.1	-	-	[38]
Tomato (crushed)	3	1.22 (1.69)	0.24–3.17	0.14 (0.02)	0.12–0.16	9.66 (8.78)	4.5–19.80	0.18 (0.06)	0.12–0.23	#
	-	2	-	2	-	20	-	-	-	[34]
Tomato (ketchup)	3	0.37 (0.64)	nd–1.11	nd	-	1.07 (0.08)	1–1.15	nd	-	#
	24	-	-	-	-	52.5 (54.1)	nd–165	-	-	[38]
	-	22	-	-	-	-	-	-	-	[12]
**Legumes and Derivatives**										
Beans (white)	6	nd	-	nd	-	0.66 (0.64)	0.35–1.96	nd	-	#
	-	-	-	2	-	3	-	-	-	[34]
Beans (red kidney)	3	-	-	-	-	-	0.3–0.4	-	-	[50]
	5	-	-	-	-	-	nd–4	-	-	[52]
	-	-	-	3	-	1	-	-	-	[34]
Chickpeas	4	nd	-	nd	-	3.63 (2.49)	0.90–6.39	nd	-	#
	-	-	-	<0.50	-	2	-	<0.50	-	[34]
Lentils	7	nd	-	nd	-	8.19 (8.36)	1.96–21.81	nd	-	#
	5	-	-	-	-	-	nd–20.2	-	-	[52]
	-	-	-	-	-	3	-	-	-	[34]
Peanuts	7	nd	-	nd	-	0.87 (1.01)	nd–2.56	nd	-	#
Peas	9	nd	-	nd	-	34.28 (13.50)	8.74–54.44	nd	-	#
	10	-	-	-	-	17.3	-	-	-	[48]
	6	-	-	-	-	32.3	5.5–51.1	-	-	[49]
Peas (frozen)	14	-	-	-	-	46.3 (27)	11.7–107	-	-	[38]
Soybean, dried	3	-	-	-	-	-	1.6–6.5	-	-	[50]
	1	-	-	-	-	17	-	-	-	[47]
	2	-	-	-	-	41	-	-	-	[51]
	13	-	-	-	-	-	3.7–16.8	-	-	[55]
	4	-	-	-	-	30.9 (15.5)	16.3–57	-	-	[52]
	2	-	-	-	-	-	35.2–57.2	-	-	[40]
	5	-	-	-	-	17.1	6.4–24.2	-	-	[49]
Soybean milk	3	nd	-	nd	-	1.02 (0.73)	0.39–1.81	0.28 (0.24)	nd–0.42	[39]
	2	-	-	-	-	2.11	-	13.9	-	[40]
Soybean sprouts	3	-	-	-	-	44.71 (3.21)	41.13–47.43	0.21 (0.18)	nd–0.33	[39]
Tofu	6	nd	-	nd	-	0.76 (0.55)	nd–1.49	0.67 (0.49)	nd–1.42	#
	4	-	-	-	-	nd	-	-	-	[49]
	19	-	-	-	-	2.6 (1.4)	nd-5	-	-	[56]
	2	-	-	-	-	1.76	-	18.4	-	[40]

Here, *n*: number of samples; SD: standard deviation; nd: not detected; -: values not reported by the study; #: data on the biogenic amine content from our own database of Spanish market products.

**Table 2:** Biogenic amine contents (mg/kg fresh weight) found in fruits and nuts. Data are presented as average (standard deviation) and range (minimum–maximum).

Food Categories	*n*	Occurrence of Biogenic Amines (mg/kg)								Reference
		Histamine		Tyramine		Putrescine		Cadaverine		
		Mean (SD)	Range	Mean (SD)	Range	Mean (SD)	Range	Mean (SD)	Range	
**Fruit and Fruit Products**										
Apple	3	-	-	-	-	-	0.4–1.7	-	-	[50]
	2	-	-	-	-	nd	-	-	-	[51]
	2	-	-	-	-	1.5	-	nd	-	[40]
Apple juice	10	nd	-	0.67 (0.50)	nd–1.6	1.02 (0.35)	0.59–1.68	2.30 (1.53)	0.55–4.27	[41]
Avocado	5	nd	-	1.81 (2.06)	0.58–5.44	nd	-	nd	-	#
	-	23	-	-	-	-	-	-	-	[12]
	2	-	-	-	-	nd	-	nd	-	[40]
Banana	8	nd	-	0.53 (0.79)	nd–1.85	37.94 (8.32)	25.50–49.49	nd	-	#
	2	-	-	-	-	-	15.86–41.05	10.83	-	[40]
Cherry	5	nd	-	nd	-	3.08 (0.51)	2.35–3.46	nd	-	#
	2	-	-	-	-	4.67	-	nd	-	[40]
Grape	2	-	-	-	-	9.34	-	5.93	-	[40]
Grapefruit	2	nd	-	nd	-	55.55 (12.8)	46.52–64.57	nd	-	#
	2	-	-	-	-	51.1	-	nd	-	[40]
Grapefruit Juice	3	-	-	-	-	98.6	-	-	-	[50]
	10	0.31 (0.58)	nd–1.74	nd	-	10.08 (4.11)	7.17–20.8	1 (0.64)	0.38–2.28	[41]
Guava	21	-	-	-	-	1	0.4–1.8	-	-	[57]
Kiwi	13	nd	-	nd	-	2.49 (3.96)	0.5–15.57	nd	-	#
	2	-	-	-	-	1.06	-	nd	-	[40]
	-	1.9 (0.1)	-	nd	-	3.1 (0.1)	-	nd	-	[54]
Lemon	3	nd	-	nd	-	2.33 (2.02)	nd–3.67	nd	-	#
Mandarin	21	nd	-	0.94 (1.31)	nd–5.76	90.16 (36.6)	12.29–173.8	nd	-	#
	10	-	-	-	-	122 (44.2)	67.3–200	-	-	[42]
Mango	21	-	-	-	-	0.9	nd–2.7	-	-	[57]
Orange	12	nd	-	nd	-	91.24 (41.7)	11.34–151.1	nd	-	#
	3	-	-	-	-	-	95.1–140	-	-	[50]
	2	-	-	-	-	117	-	-	-	[51]
	5	-	-	-	-	137 (11.3)	119–153	-	-	[42]
	2	-	-	-	-		54.62–119.82	2.04	-	[40]
Orange juice	3	nd	-	nd	-	45.51 (10.5)	37.35–57.3	nd	-	#
	3	-	-	-	-	85 (11.4)	76.6–100	-	-	[42]
	11	0.46 (0.41)	nd–1.32	nd	-	45.51 (8.35)	34.70–60.97	nd	-	[41]
Papaya	21	-	-	-	-	11	5.3–19.3	-	-	[57]
	2	-	-	-	-	4.67	-	nd	-	[40]
Passion fruit	21	-	-	-	-	17.9	6.5–40.5	-	-	[57]
Pear	3	-	-	-	-	-	23.6–24.2	-	-	[50]
	2	-	-	-	-	1.5	-	0.41	-	[40]
Peach	2	nd	-	nd	-	1.92 (0.14)	1.82–2.02	nd	-	#
	2	-	-	-	-	0.35	-	<0.10	-	[40]
Pineapple	6	nd	-	nd	-	4.20 (2.17)	1.39–7.96	nd	-	#
	2	-	-	-	-	4.05	-	3.07	-	[40]
	21	-	-	-	-	1.1	nd–2.5	-	-	[57]
Pineapple juice	12	2.44 (1.59)	nd–4.61	0.87 (0.86)	nd–1.93	1.79 (0.16)	1.53–1.98	1.21 (1.22)	nd–3.14	[41]
Plum	2	nd	-	4.02 (4.32)	0.96–7.07	nd	-	nd	-	#
Strawberry	9	nd	-	nd	-	3.77 (1.52)	2.04–6.42	nd	-	#
	2	-	-	-	-	0.97	-	4.29	-	[40]
**Nuts**										
Almonds	7	nd	-	nd	-	2.47 (1.24)	nd–4.36	nd	-	#
	2	-	-	-	-	4.32	-	5.57	-	[40]
Chestnuts	2	nd	-	nd	-	4.53 (3.40)	2.12–6.93	nd	-	#
	2	-	-	-	-	5.2	-	1.33	-	[40]
Hazelnuts	9	nd	-	0.49 (0.85)	nd–2.63	1.18 (1.09)	nd–3.19	nd	-	#
Nuts	6	nd	-	nd	-	5.64 (4.17)	2.82–13.79	nd	-	#
Pistachios	7	nd	-	nd	-	14.84 (14.0)	4.31–39.51	1.65 (4.37)	nd–11.58	#
	2	-	-	-	-	43	-	3.27	-	[40]
Sunflower seeds	2	nd	-	nd	-	0.50 (0.19)	0.36–0.63	nd	-	#
	2	-	-	-	-	3	-	22.58	-	[40]

Here, *n*: number of samples; SD: standard deviation; nd: not detected; -: values not reported by the study; #: data on the biogenic amine content from our own database of Spanish market products.

**Table 3:** Biogenic amine contents (mg/kg fresh weight) found in cereals and cereal-based products. Data are presented as average (standard deviation) and range (minimum–maximum).

Food Categories	*n*	Occurrence of Biogenic Amines (mg/kg)								Reference
		Histamine		Tyramine		Putrescine		Cadaverine		
		Mean (SD)	Range	Mean (SD)	Range	Mean (SD)	Range	Mean (SD)	Range	
Barley	2	nd	-	nd	-	2.19 (1.55)	1.09–3.28	nd	-	#
Bread, white	2	nd	-	nd	-	nd	-	nd	-	#
	3	-	-	-	-	-	1.5–1.8	-	-	[50]
	10	-	-	-	-	1.1	-	-	-	[48]
	2	-	-	-	-	1.32	-	2.35	-	[40]
Bread, wholemeal	6	nd	-	nd	-	1.96 (1.45)	nd–4.32	nd	-	#
									
Cereal (corn, chocolate)	8	nd	-	nd	-	0.32 (0.44)	nd–0.93	nd	-	#
	10	-	-	-	-	-	2-2.2	-	-	[50]
Oats	2	nd	-	nd	-	0.67 (0.32)	0.44–0.89	nd	-	#
Pasta (wheat)	7	nd	-	nd	-	1.56 (1.65)	0.81–4.52	nd	-	#
Rice	2	nd	-	nd	-	2.4 (0.03)	2.38–2.42	nd	-	#
	2	-	-	-	-	<0.9	-	-	-	[51]
	2	-	-	-	-	1.2	-	-	-	[40]
	6	-	-	-	-	0.2	0.2–0.3	-	-	[49]
	10	-	-	-	-	0.2	-	-	-	[48]
Wheat germ	2	nd	-	nd	-	31.64 (0.35)	31.39–31.9	0.63 (0.08)	0.57–0.69	#
	2	-	-	-	-	62.1	-	-	-	[40]

Here, *n*: number of samples; SD: standard deviation; nd: not detected; -: values not reported by the study; #: data on the biogenic amine content from our own database of Spanish market products.

**Table 4:** Content of histamine and other biogenic amines (mg/kg fresh weight) in plant-origin foods excluded from different low-histamine diets [1,2,3,4,5,6,7,8]. Data obtained from own database and from different scientific studies [5,12,34,38,39,40,41,42,47,48,49,50,51,52,53,54,55,57].

Food Items	Histamine	Putrescine	Cadaverine	Tyramine	Spermidine	Spermine
Spinach ^a^	9–70	nd–119	nd–9	1–10	14–53	nd–9
Eggplant ^a^	4–101	24–49	nd–5	nd–2	2–12	nd–6
Tomato ^a^	nd–17	5–122	nd–2	nd–6	2–16	nd–2
Ketchup ^a^	nd–22	nd–165	nd	nd	nd–33	nd–12
Avocado ^a^	nd–23	nd	nd	0.5–5	nd–7	2–8
Citrus (fresh and juices) ^b^	nd–2	7–200	nd–2	nd–5	nd–12	nd–5
Mushroom ^b^	nd	nd–156	nd	nd	9–155	nd–13
Banana ^b^	nd	15–50	nd–10	nd–2	8–16	nd–3
Soybean or soybean sprouts ^b^	nd	2–57	nd–0.3	nd	33–389	7–114
Nuts ^b^	nd	nd–40	nd–23	nd–3	6–40	2–33
Pears ^b^	-	2–25	nd–0.4	-	30–76	8–49
Lentils ^b^	nd	nd–21	nd	nd	15–107	5–18
Chickpeas ^b^	nd	1–6	nd–0.5	nd–0.5	15–85	4–32
Peanuts ^c^	nd	nd–3	nd	nd	23–48	5–13
Kiwi ^c^	nd–2	nd–15	nd	nd	3–6	nd–2
Papaya ^c^	-	5–20	nd	-	4–8	nd–2
Strawberry ^c^	nd	2–6	nd–4	nd	5–10	nd–2
Pineapple ^c^	nd	nd–8	nd–3	nd	nd–3	nd–1
Plum ^c^	nd	nd	nd	1–7	2–3	nd–4

Here, nd: not detected; -: values not reported by the studies; ^a^ plant-origin foods with histamine; ^b^ plant-origin foods without histamine but with high contents of other amines; ^c^ plant-origin foods with low levels of all amines.
